# Preparation of Blood-Deficient Model and Research of Angelica Polysaccharide on Enriching Blood in Chickens

**DOI:** 10.1155/2012/965947

**Published:** 2012-05-22

**Authors:** Haifeng Hou, Yongzhan Bao, Qian Li, Wanyu Shi

**Affiliations:** ^1^College of Veterinary Medicine, Agricultural University of Hebei, Baoding 071001, China; ^2^Department of Animal Husbandry and Veterinary Medicine, Baoding Vocational and Technical College, Baoding 071051, China; ^3^Egg-Type Chicken Laboratory, Animal Husbandry and Veterinary Institute of Hebei, Baoding 071000, China

## Abstract

In this study cyclophosphamide was used to prepare the blood-deficient model. The red blood cell count and hemoglobin content were measured. The experimental chickens presented the symptoms of blood-deficient syndrome, dullness, shrinkinginto oneself, broken winded, loose feather, waxy eyelid, and pale tongue. At the same time, red blood cell count and hemoglobin content decreased significantly. Angelica polysaccharide as the effective component of Angelica Sinensis could significantly increase the red blood cell count and the hemoglobin content of blood-deficient chickens. The results indicated that cyclophosphamide could significantly reduce the red blood count and hemoglobin content, and make the ideal blood-deficient model successfully. Angelica polysaccharide had the function of enriching blood in different ways. On the one hand Angelica polysaccharide enriched he blood directly, increased the number of RBC and hemoglobin; on the other hand it regulated the hematopoietic factors, enriched the blood indirectly.

## 1. Introduction

Based on the traditional Chinese veterinary medicine, syndrome of blood deficiency is a morbid condition of insufficient blood supply to the visceral organs and channels for their nutrition due to improper feeding, malnutrition, profuse bleeding, chronic hemorrhage, or diminished production of blood on account of hypofunction of the internal organs. The main manifestations are pale tongue, dullness, sparse hair, thready-weak pulse, and so forth [1]. Animal experimental study is an indispensable way to illustrate the etiology and pathogenesis of blood-deficient syndrome. Likewise, animal experiment is a method to study the mechanism of hematinics on treating deficiency of blood. An animal model of blood-deficient syndrome is an important step in the research on the modernization of traditional Chinese veterinary medicine [2]. Angelica Sinensis, known as Danggui in China, is one of the most popular traditional Chinese medicines as blood-enriching drugs. The medicinal part is composed of its dried roots with sweet-acrid taste, warm property, and attribution to the heart, liver and spleen channels. It is commonly used to enrich or nourish blood and activate blood circulation. Angelica Sinensis is contained by more than 80 composite formulae. Modern researches indicate that phthalides, organic acids, and their esters, polysaccharides are the main chemical components related to the bioactivities and pharmacological properties of Danggui [3–5]. Angelica polysaccharide is water-soluble organic compound extracted from the root of Angelica Sinensis. Angelica polysaccharide, as the effective component of Angelica Sinensis in enriching blood, has the efficacy of purifying blood quality, emmenagogue, acesodyne, lenitive, improving circulation and immunity, antiviral, antitumor, antioxidation, and so forth [6–9]. It is used frequently in clinics and also frequently appears as the main ingredient in prescriptions for bone injures. Cyclophosphamide is a cytostatic agent that produces systemic toxicity especially on cells with high proliferative capacity, while polysaccharides from Angelica polysaccharide have been shown to increase the turnover of hemopoietic stem cells [10]. In the modern society Angelica polysaccharide has a better potential for drug development [11]. Previous research showed Angelica polysaccharide had effects on enriching blood in mice/human with blood deficiency [10, 12]. However, there's less reports on its hematopoiesis of Angelica polysaccharide about poultry. In this study, Cyclophosphamide was used to investigate whether the blood-deficient model was successfully made in chickens, at the same time to discuss the mechanisms on enriching and nourishing blood of Angelica polysaccharide [12–16].

## 2. Materials and Methods

### 2.1. Animals

One-day-old Hyline Brown chickens (male) were purchased from Hebei laboratory animal center, housed in cages and lighted for 24 h at the beginning of pretrial period. The chicks were given free access to feedstuff and water.

### 2.2. Reagent

Angelica polysaccharide was purchased from Beijing Biochem Co., Ltd. China. Cyclophosphamide was purchased from Jiangsu Hengrui Medicine Co., Ltd (no. 06121021).

### 2.3. Grouping and Treatment

Eighty 14-day-old normal chickens were randomly divided into eight groups with the same number and similar body weight. Angelica polysaccharide (1%) diluted with distilled water was drenched to the chickens by oral administration in a sterile syringe without a needle. The healthy chickens in group I as control, the healthy chickens in group II, III, and IV were given three gradient dosages (50 mg/Kg, 100 mg/Kg, 150 mg/Kg) of Angelica polysaccharide, respectively, for 7 days. Chickens in group V, VI, VII, and VIII were given Cyclophosphamide by intraperitoneal injection, 6 days later the blood-deficient chicken model was made. After the blood-deficient chicken model was set up, chickens in group VI, VII, and VIII were given three gradient dosages (50 mg/Kg, 100 mg/Kg, 150 mg/Kg) of Angelica polysaccharide, respectively, for 7 days. All the experimental animals were treated in accordance with the guidelines of the Chinese Council for Animal Care.

### 2.4. Blood Specimen Collection and Examination

Until the last Angelica polysaccharide administration, the blood specimens were collected from heart for red blood cells (test tube method) and hemoglobin (turbidimetry) tests. The steps were followed with [17].

### 2.5. Data Statistics

All data had a normal distribution presented as mean ± standard deviation (SD) and analysed by SPSS11.0 statistical software. Statistical significance was determined by one-way analysis of variance (ANOVA) followed by student's *t*-test. A probability of less than 0.01 was considered to be statistically significant.

## 3. Results

### 3.1. Symptoms

The blood-deficient model was made by intraperitoneal injection of Cyclophosphamide for 6 days (80 mg/Kg·d). 6 days later, the chickens behaved as follows: dullness, shrinking into oneself, broken winded, loose-feather, waxy eyelid, and pale tongue. It can be concluded from the clinical manifestations that chicken for blood-deficient syndrome model has been successfully set up. Healthy chickens given Angelica polysaccharide behaved as follows, physical agility, pink tongue and bright feather. The blood-deficient chickens returned to the normal symptoms after given Angelica polysaccharide. Blood-deficient chickens given Angelica polysaccharide had no notable changes than the control group.

### 3.2. Effects of Angelica Polysaccharide on Blood Physiological Index

#### 3.2.1. Effects of Angelica Polysaccharide on the Red Blood Cell Count

It showed in Table 1 that the red blood cell count in group (II) had no more change than the control group (*P* > 0.05). Angelica polysaccharide in group (III) and (IV) had significant effects on the red blood cell count than group I (*P* < 0.01). When the healthy chickens were given Cyclophosphamide, there was a significant decrease in the number of erythrocyte in group V (*P* < 0.01). However, after the blood-deficient chickens were given Angelica polysaccharide, the number of erythrocyte gradually increased. In added, the red blood cell count in group VIII was notably higher than the control (*P* < 0.05). The number of erythrocyte in group VII and VIII were significantly higher than group V (*P* < 0.01). In a word, Cyclophosphamide can lead to the red blood cell count reduction and Angelica polysaccharide as hematinic can increase it.

#### 3.2.2. Effects of Angelica Polysaccharide on Hemoglobin Content

It showed in Table 2 that hemoglobin content in group II had no more change than the control group (*P* > 0.05). Angelica polysaccharide in group III and IV had significant effects on hemoglobin content than group I (*P* < 0.01). When the healthy chickens were given Cyclophosphamide, there was a significant decrease in the hemoglobin content in group V (*P* < 0.01). However, after the blood-deficient chickens were given Angelica polysaccharide, the hemoglobin content gradually increased. The hemoglobin content in group VII and VIII were significantly higher than group V (*P* < 0.01). In a word, Cyclophosphamide can lead to the hemoglobin content reduction and Angelica polysaccharide as hematinic can increase it.

## 4. Discussion

At present many methods which have different characteristics could set up blood-deficient animal model, such as radiation damage method, chemical method, and immune induce method [18]. Bleeding method which does not use special equipment is simple, with definite index. Bleeding method brings about the decrease of the peripheral blood cells immediately; however, the bloodletting quantity is hard to control and has little effect on the hematopoietic system of organism [19]. Radiation damage method which uses 60Co-*γ* to irradiate animals could damage bone marrow and affect the hematopoietic function. Radioactive ray has direct damage to stem cells and bone marrow microenvironment. Radioactive ray brings down the bone marrow and affects CFU-E, BFU-E, and CFU-GM at certain dosage. Radiation damage method needs to use special equipment, at the same time the radiation dosage is difficult to control. When the radiation dosage is low it cannot meet the damage requirement. Likewise, if the radiation dosage is high it may lead to death [20].

As the chemical method, in this experiment Cyclophosphamide was used to make the blood-deficient model [21]. The dosage, route and times of Cyclophosphamide administration were all easily to control by intraperitoneal injection. Cyclophosphamide is a kind of nitrogen mustards alkylating agent produced synthetically. Cyclophosphamide which is a broad spectrum anti-tumor medicine is widely used to treat acute/chronic lymphocytic leukemia, malignant lymphoma, myelomatosis multiplex, and so forth. When Cyclophosphamide got into animal body, it broke down into chloroethyl phosphopeptamine with alkylating function induced by hepatomicrosome P450 enzyme system. Then chloroethyl phosphopeptamine led to single-strand and double-strand DNA broken by cross-linking DNA strands. As an antitumor drug, Cyclophphosphamide destroyed the structure of DNA directly, interfered the transcription of DNA, inhibited the synthesis of RNA and protein, thus Cyclophosphamide prevented the proliferation of cell and reduced the blood supply. So Cyclophosphamide caused widespread destruction of hemopoietic system and immune system [22, 23]. In clinic, the effects of Cyclophosphamide are performed mainly in immunological function repression, marrow inhibition and peripheral blood cell decrease [24, 25]. Experiments indicated Cyclophosphamide had obvious inhibitive effects on red cell immune function [26]. Studies showed that Cyclophosphamide could also damage the bone marrow microenvironment and the proliferation and differentiation of hematopoietic cell, in order to inhibit the hematopoietic function [23, 27]. The two inhibitive effects made the deficiency of blood.

In order to research the pathophysiological changes of blood-deficient syndrome, experimental index interrelated with Ying blood function was chosen to examine [28]. Erythrocyte and hemoglobin could reflect the quantitative and functional changes of blood-deficient syndrome. The red blood cell count and hemoglobin content are both important standards to measure. Clinically, blood loss and anemia often present with the reduction of erythrocyte and hemoglobin content [29–31]. Angelica Sinensis as the traditional Chinese medicine is one of the most popular medicine to treat blood-deficient syndrome, and Angelica polysaccharide is the major component of Angelica Sinensis in enriching blood. Recent studies demonstrated that Angelica polysaccharide could effect the hemopoietic system of animal and had obvious promoter action on proliferation and differentiation of myelogenous hemopoietic progenitor cell of human and rats. It was also reported that indicated Angelica polysaccharide could increase C3b receptor rates, leucocyte and thrombocyte in peripheral blood of mice with radiation-injury remarkably. Likewise, Angelica polysaccharide could improve the hemopoietic function of radiation injured mice [32]. Studies showed that supernatant induced by Angelica polysaccharide of bone marrow macrophage could increase the colony-forming efficiency of myelogenous hemopoietic progenitor cell. Induced by Angelica polysaccharide, protein levels of EPO, GM-CSF, IL-3, and IL-6 expressed by bone marrow macrophage were improved differently, at the same time the expression levels and strength of mRNA of EPO and GM-CSF were increased significantly [33]. At the gene level and protein level, Angelica polysaccharide promoted the synthesis and excretion hemopoiesis regulatory factors and then promoted the proliferation and differentiation of hemopoietic progenitor cell. It can be seen that Angelica polysaccharide not only supports the normal hemopoiesis but also inhibits the proliferation of tumor cell such as leukemia. It can be used for a natural revulsant. The hematopoietic function of Angelica polysaccharide works in different ways. On the one hand Angelica polysaccharide enriches he blood directly, increases the number of RBC and hemoglobin; on the other hand it regulates the hematopoietic factors, enriches the blood indirectly.

In this experiment the results indicated that Angelica polysaccharide could improve the symptoms of blood-deficient syndrome made by Cyclophosphamide, at the same time, significantly increase the red blood cell count and the hemoglobin content of blood-deficient chickens. With the development of scientific and technological and traditional Chinese medicine theory, mechanism of Angelica polysaccharide on enriching blood will be deepened and systematized gradually.

## 5. Conclusion

This study suggests that Cyclophphosphamide can make the ideal blood-deficient model successfully by intraperitoneal injection for 6 days (80 mg/Kg·d) when the chickens were 14-day-old. The experimental chickens presented the symptoms of blood-deficient syndrome, dullness, shrinking into oneself, broken winded, loose feather, waxy eyelid, and pale tongue. At the same time, red blood cell count and hemoglobin content decreased significantly. This blood-deficient model remains the advantage of high survival rate as well as long duration of blood-deficient symptoms. The results also show that Angelica polysaccharide can significantly increase the red blood cell count and hemoglobin content of blood-deficient chickens.

In conclusion, the blood-deficient model made by intraperitoneal injection of Cyclophphosphamide is more suitable for the clinical manifestations. As the main component of Angelica Sinensis, Angelica polysaccharide has the function of enriching blood. The finding provides a better basis for the clinic use of hematopoietic.

## Figures and Tables

**Table 1:** Effects of Angelica polysaccharide on red blood cell count in chickens.

Group	*N*	Dosage (mg/kg)	Count of RBC (×10^12^/L)
(I) Control of healthy chicken	10	0	276.453 ± 23.412^△△^
(II) Low dosage in healthy chicken	10	50	279.579 ± 25.890
(III) Middle dosage in healthy chicken	10	100	312.132 ± 12.900**
(IV) High dosage in healthy chicken	10	150	313.256 ± 17.203**
(V) CY	10	0	241.557 ± 14.498**
(VI) CY with low dosage	10	50	262.624 ± 15.560
(VII) CY with middle dosage	10	100	283.233 ± 13.412^△△^
(VIII) CY with high dosage	10	150	298.684 ± 21.287^∗△△^

**Superscript differs significantly (*P* < 0.01) compared with group I, *superscript differs notably (*P* < 0.05) compared with group I. ^△△^Superscript differs significantly (*P* < 0.01) compared with group V, ^△^superscript differs notably (*P* < 0.05) compared with group V. CY stands for Cyclophosphamide. RBC stands for red blood cell.

**Table 2:** Effects of Angelica polysaccharide on hemoglobin content in chickens.

Group	*N*	Dosage (mg/kg)	Hemoglobin content (g/L)
(I) Control of healthy chicken	10	0	71.203 ± 9.898^△△^
(II) Low dosage in healthy chicken	10	50	77.316 ± 2.493
(III) Middle dosage in healthy chicken	10	100	84.611 ± 3.752**
(IV) High dosage in healthy chicken	10	150	86.684 ± 5.610**
(V) CY	10	0	63.797 ± 3.337**
(VI) CY with low dosage	10	50	72.541 ± 5.630
(VII) CY with middle dosage	10	100	78.255 ± 7.266^△△^
(VIII) CY with high dosage	10	150	78.256 ± 3.538^△△^

**Superscript differs significantly (*P* < 0.01) compared with group I, *superscript differs notably (*P* < 0.05) compared with group I. ^△△^Superscript differs significantly (*P* < 0.01) compared with group V, ^△^superscript differs notably (*P* < 0.05) compared with group V. CY stands for Cyclophosphamide.
